# The Role of Traditional Chinese Medicine in the Regulation of Oxidative Stress in Treating Coronary Heart Disease

**DOI:** 10.1155/2019/3231424

**Published:** 2019-02-24

**Authors:** Xinyu Yang, Tianmai He, Songjie Han, Xiaoyu Zhang, Yang Sun, Yanwei Xing, Hongcai Shang

**Affiliations:** ^1^Key Laboratory of Chinese Internal Medicine of the Ministry of Education, Dongzhimen Hospital Affiliated to Beijing University of Chinese Medicine, Beijing, China; ^2^Guang An Men Hospital, Chinese Academy of Chinese Medical Sciences, Beijing, China

## Abstract

Oxidative stress has been closely related with coronary artery disease. In coronary heart disease (CHD), an excess of reactive oxygen species (ROS) production generates endothelial cell and smooth muscle functional disorders, leading to a disequilibrium between the antioxidant capacity and prooxidants. ROS also leads to inflammatory signal activation and mitochondria-mediated apoptosis, which can promote and increase the occurrence and development of CHD. There are several kinds of antioxidative and small molecular systems of antioxidants, such as *β*-carotene, ascorbic acid, *α*-tocopherol, and reduced glutathione (GSH). Studies have shown that antioxidant treatment was effective and decreased the risk of CHD, but the effect of the treatment varies greatly. Traditional Chinese medicine (TCM) has been utilized for thousands of years in China and is becoming increasingly popular all over the world, especially for the treatments of cardiovascular diseases. This review will concentrate on the evidence of the action mechanism of TCM in preventing CHD by modulating oxidative stress-related signaling pathways.

## 1. Introduction

Coronary heart disease (CHD) is one of the primary reasons of death in the world, with 7.4 million deaths in 2013, being responsible for one-third of all deaths [1–3]. By 2020, it is forecasted that CHD will continue to be the prime and most prevalent threat to human life [4]. CHD is multifactorial and concerns intricate interactions between physiological, genetic, and lifestyle factors [5]. In past studies, traditional risk factors of CHD like diabetes, hypertension, smoking, and hyperlipidemia are linked with oxidative stress [6–8]. However, a number of studies have also associated oxidative stress with the mechanism of coronary atherosclerosis and have assessed the markers of oxidative stress, indicating that they can predict the occurrence of CHD [9]. Therefore, oxidative stress is one of the risk factors of CHD, which can affect the prognosis and reduce the survival time and quality of life of patients with CHD [10, 11].

Oxidative stress has been closely related with the mechanism of atherosclerosis and coronary artery disease. Oxidative stress may take place when the antioxidant ability is insufficient to decrease reactive oxygen species (ROS) and other free radicals. When oxidative stress occurs, ROS may generate oxidative modification or lipid peroxidation damage at the deoxyribonucleic acid (DNA) level and protein level with harmful consequences for the structure and function of the vascular system [12, 13]. In CHD, microvascular pathology revealed a higher level of ROS. The production of excess ROS generates endothelial cells and smooth muscle functional disorder, leading to a disequilibrium between the antioxidant capacity and prooxidants, thus leading to inflammatory signal activation and mitochondria-mediated apoptosis, which can promote and increase the occurrence and development of CHD [14, 15].

**Figure 1:**
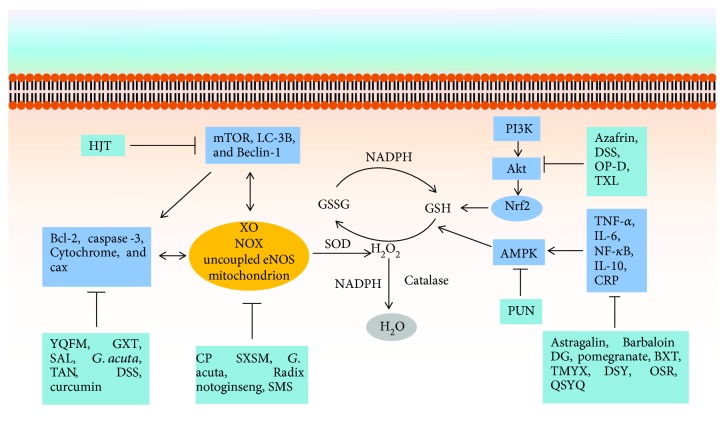
The mechanism of TCM in preventing CHD by oxidative stress-related signaling pathways. HJT: Hongjingtian injection; mTOR: mammalian target of rapamycin; LC-3B: light chain 3B; NADPH: nicotinamide adenine dinucleotide phosphate; GSSG: glutathione disulfide; GSH: glutathione; SOD: superoxide dismutase; PI3K: phosphoinositide 3-kinase; Akt: serine/threonine kinase; Nrf2: nuclear factor erythroid-2-related factor 2; AMPK: adenosine monophosphate-activated protein kinase; PUN: punicalagin; DSS: Danshensu; OP-D: Ophiopogonin D; TXL: Tongxinluo; TNF-*α*: tumor necrosis factor-*α*; IL-6: interleukin-6; NF-*κ*B: nuclear factor-*κ*B; IL-10: interleukin-10; CRP: C-reaction protein; DG: Dunye Guanxinning; TMYX: Tongmai Yangxin pill; DSY: Dan-Shen-Yin; OSR: oxysophoridine; QSYQ: Qi-shen-yi-qi; XO: xanthine oxidase; NOX: NADPH oxidase; eNOS: endothelial nitric oxide synthase; CP: cardiotonic pill; SXSM: Shenxian-shengmai; SMS: Shengmai San; Bcl-2: B-cell lymphoma-2; Bax: Bcl-2-associated protein X; YQFM: YiQiFuMai powder injection; SAL: salvianolic acid; TAN: tanshinone; GXT: Guanxintai; BXT: Bao-Xin-Tang; *G. acuta*: *Gentianella acuta*.

There are several types of vital antioxidative systems, including superoxide dismutase (SOD), catalase (CAT), and glutathione peroxidase (GSH-PX). There are also numerous crucial small molecular antioxidants such as *β*-carotene, ascorbic acid, *α*-tocopherol, and reduced glutathione (GSH) [16]. Studies have shown that antioxidant treatment was effective and decreased the risk of CHD [17]. Oxidative status records for a particular patient are generally inadequate, and specific antioxidants suitable for that patient are rarely prescribed [18] which influences treatment effectiveness. However, traditional Chinese medicine (TCM) has been utilized for thousands of years in China and is becoming increasingly popular all over the world, especially for the treatment of cardiovascular diseases [19]. Modern pharmacological research has indicated that many Chinese herbal extracts protect the development of cardiovascular diseases through their antioxidating effects [20–22]. A schematic diagram of the mechanisms of ROS is demonstrated in Figure 1. This review will concentrate on the current evidence of the action mechanism of TCM in preventing CHD by modulating oxidative stress-related signaling pathways.

## 2. Protective Effects of Traditional Chinese Medicine (TCM) and Its Constituent Compounds on Coronary Heart Disease

### 2.1. Myocardial Infarction (MI)

MI is considered as one of the most common forms of ischemic heart disease and is one of the main reasons of death worldwide. A growing body of evidence has indicated that ROS can lead to cell loss following MI and is closely related to the generation of MI [23]. ROS reduction may represent a vital therapeutic target for relieving the damage caused by a MI. Therefore, targeting the production of ROS with all kinds of antioxidants has been shown to decrease oxidative stress-related injury and therefore improve MI status.

#### 2.1.1. The Bioactive Ingredients of Traditional Chinese Medicine

*Salvia miltiorrhiza: Salvia miltiorrhiza*, a famous Chinese herb medicine, has been widely used in treating cardiovascular diseases [24]. Studies showed that it could relieve small artery circulation, decrease ROS production [25–27], restrain cell apoptosis [27–29], and protect the heart against ischemia-reperfusion injury [30–32]. *Salvianolic acid* (SAL, C_36_H_30_O_16_; C_26_H_22_O_10_) and *tanshinone* (TAN, C_18_H_12_O_3_; C_19_H_18_O_3_), hydrophilic and lipophilic compounds, are extracted from *Salvia miltiorrhiza* [33]. Wang et al. [34] studied the use of the MI models to evaluate the cardioprotective functions of SAL and TAN in rats. Both echocardiographic and infarct sizes were assessed after surgery, while gene activity was detected by microarray analysis and validated by quantitative real-time reverse transcription-polymerase chain reaction (RT-PCR). These results depicted that SAL is possibly mediated by the downregulation of factors participating in oxidative stress and apoptosis, while TAN is probably mediated by the suppression of intracellular calcium and cell adhesion pathways in the MI.

Danshensu (DSS, C_9_H_10_O_5_), the main water-soluble ingredient of *Salvia miltiorrhiza*, has also been studied as a significant compound. In a study [35], DSS was detected in an ischemia-reperfusion (I/R) model to research its cardioprotective function. The results showed that DSS significantly reduced the level of creatine kinase and lactate dehydrogenase and that DSS had ROS scavenging activity and enhanced endogenous antioxidants such as SOD, CAT, malondialdehyde (MDA), GSH-PX, and heme oxygenase-1 (HO-1) activities through stimulation of the nuclear factor erythroid-2-related factor 2 (Nrf2) signaling pathway which was regulated by serine/threonine kinase (Akt) and extracellular signaling-regulated kinase 1/2 (ERK1/2) signaling pathway in a western blot analysis. The mechanism might be associated with the improvement of the antioxidant defense system by stimulating Akt/ERK1/2/Nrf2 signaling pathways [36, 37].

*Astragalin: astragalin* (C_21_H_20_O_11_) is a flavonoid that is extracted from the leaves of *Rosa agrestis*, *persimmon*, or green tea seeds. A large number of studies have indicated that *astragalin* has wide pharmacological activities, covering anti-inflammatory, antioxidative, and other beneficial activities [38–40]. A study [41] that assessed the cardioprotective functions of *astragalin* against I/R injury in the rat heart by Langendorff apparatus has been conducted. The results revealed that *astragalin* pretreatment ameliorated myocardial function. SOD activity and the glutathione/glutathione disulfide (GSH/GSSG) ratio were dramatically enhanced, and the levels of MDA, tumor necrosis factor-*α* (TNF-*α*), intracellular ROS, and interleukin-6 (IL-6) were reduced in the *astragalin*-treated groups. Thus, *astragalin* displayed cardioprotective functions through its antiapoptotic, antioxidative, and anti-inflammatory activities [41–43].

*Ophiopogonin D (OP-D): OP-D* (C_44_H_70_O_16_) is a significantly valid monomeric ingredient used in the Shenmai injection (SM-I). It is reported that it has a wide range of biological activities, including antiapoptotic effects, antioxidant, and anti-inflammatory actions [44–47]. The rat model of myocardial ischemia-reperfusion (MI/R) damage was produced by ligation of the left anterior descending coronary artery to study the protective actions and underlying mechanisms of *OP-D* and SM-I [48]. The study found that *OP-D* and SM-I act by inducing cardioprotection on MI/R injury by regulating cardiac function, reducing acetate dehydrogenase and creatine kinase (CK) generation, decreasing infarct size, and improving the injured cardiac structures. Cardioprotection by *OP-D* and SM-I was mediated by activating the phosphoinositide 3-kinase (PI3K)/Akt/endothelial nitric oxide synthase (eNOS) signaling pathway and suppressing the nuclear factor-*κ*B (NF-*κ*B) signaling pathway [49, 50].

*Curcumin: curcumin* (C_21_H_20_O_6_), used both as a seasoning and a traditional medicine, is a natural compound derived from the roots of *Curcuma longa L*. It has various pharmacological activities such as anti-inflammatory, antioxidant, and anticarcinogenic activities in different models [51–53]. In a study [54], the possible protective function of *curcumin* on cardiac function in MI/R rats was researched. The rats suffered from myocardial injuries through ligation of the left anterior descending coronary artery. Afterwards, lipid peroxidation products and antioxidant enzymes were evaluated in the myocardial tissue. The result showed that *curcumin* might decrease the venture of coronary heart disease via activating the JAK2/STAT3 signal pathway, reducing oxidative damage and suppressing myocardium apoptosis [55–57].

*Punicalagin (PUN):* PUN (C_48_H_28_O_30_), a main bioactive constituent in pomegranate juice, has been tested for neuroprotective functions against cerebral ischemia-reperfusion (I/R) injury by antioxidative mechanisms [58, 59]. Another study [60] investigated if PUN offers cardioprotective effects against MI/R damage and the potential mechanisms. MI/R was achieved by ligating the left anterior descending coronary artery. PUN acts by ameliorating cardiac function and infarct size, decreasing serum creatine kinase-MB (CK-MB) and lactate dehydrogenase, and inhibiting myocardial apoptosis against MI/R damage. These results showed that PUN protects against I/R-induced ROS and myocardial damage by activating adenosine monophosphate-activated protein kinase (AMPK) [60, 61].

*Barbaloin (BAR):* BAR (C_21_H_22_O_9_) is the major medicinal ingredient of *Aloe vera* belonging to the liliaceous plant group that has good antioxidant properties [62]. Zhang et al. [63] investigated if BAR offers cardioprotection in the MI myocardial damage. BAR was intragastrically administrated to rats before MI operation. The result showed that BAR pretreatment efficiently suppressed I/R-induced ROS and inflammatory effects by activating AMPK signaling in MI/R rat hearts [62–65].

*Oxysophoridine (OSR):* OSR (C_15_H_24_N_2_O_2_), a natural alkaloid from the Chinese herbal medicine *Sophora alopecuroides L.*, can play multiple pharmacological roles such as the suppression of oxidative stress and apoptosis [66, 67]. A study [68] assessed the cardioprotective effect of OSR against MI in rats. OSR decreased infarction size and the levels of myocardial enzymes, including the CK-MB, cardiac troponin T, and lactate dehydrogenase. A decreased level of MDA was noticed while increased levels of catalase, SOD, glutathione peroxidase activity, and nonenzymatic scavenger glutathione were also verified in OSR-treated rats. In addition, OSR suppressed the activities of various inflammatory cytokines [69, 70]. The results showed that OSR relieves myocardial damage in the rat model of acute myocardial infarction (AMI) and that the cardioprotective effects may be associated with antiapoptotic, anti-inflammatory, and antioxidative mechanisms.

*Gentianella acuta (G. acuta): G. acuta* (C_13_H_8_O_2_) is extensively used for the therapy of coronary heart disease in Mongolian medicine. It is commonly known as “Wenxincao” in traditional Chinese medicine [71]. The potential protective effect of *G. acuta* on myocardial I/R injury by using the Langendorff apparatus in isolated rats was studied [72]. Some hemodynamic parameters were logged during the perfusion. These results showed that the *xanthones* from *G. acuta* dramatically ameliorated myocardial function and enhanced the levels of SOD, succinate dehydrogenase (SDH), CAT, malate dehydrogenase (MDH), adenosine triphosphate (ATP), and the proportion of GSH/GSSG while inhibiting the levels of CK, MDA, and LDH. Moreover, *xanthones* could upregulate the Bcl-2 protein and downregulate the Bax protein. In short, *xanthones* from *G. acuta* displayed a cardioprotective effect on myocardial I/R damage via antioxidative and antiapoptosis activities [73–76].

*Azafrin: Centranthera grandiflora Benth.* is an ethnic drug known as Ye-Can-Dou-Gen (YCDG) and has been extensively used to cure cardiovascular system diseases in China. *Azafrin* (C_27_H_38_O_4_), a carotene antioxidant, is one of the richest active compounds in YCDG [77]. Yang et al. [78] investigated the cardioprotective capacity of *azafrin* on the MI and MI/R damage to understand its potential myocardium preservation mechanisms. By experimental procedures, the results indicated that *azafrin* treatment significantly ameliorated heart function and infarct size in rats; reduced the levels of myocardial enzymes, cardiac troponin l (cTnI), and MDA; and increased SOD activity in vivo. In a word, *azafrin* displayed cardioprotective effects against myocardial damage through activation of the Nrf2-antioxidant response element (ARE) pathway [79].

#### 2.1.2. Traditional Chinese Medicine Decoction

*Bao-Xin-Tang (BXT):* BXT is a Chinese herbal compound used to treat coronary heart disease and is made of *Codonopsis pilosula*, *Atractylodes macrocephala*, *Astragalus*, *Fructus crataegi*, etc. Previous studies have verified that it can ameliorate blood circulation to protect the myocardium of patients with MI [80]. A study [81] designed to explore if BXT offers cardioprotection against MI has been conducted. The rat model of MI was made by the ligation of the left anterior descending coronary artery. The data suggested that BXT could decrease the infarction size, myeloperoxidase, interleukin-6 (IL-6), and levels of C-reactive protein (CRP) and enhance SOD activities and anti-inflammatory media such as interleukin-10 (IL-10). Thus, the functions of BXT may be associated with antioxidant and anti-inflammation properties [82, 83].

*Dan-Shen-Yin (DSY):* DSY, including sandalwood *Fructus amomi* and *Salvia miltiorrhiza*, is a famous Chinese herbal formula which is extensively used for the therapy of CHD [84, 85]. A study [86] explored whether DSY could protect from MI. The left anterior descending branch of the coronary artery was ligated to induce myocardial ischemia in rats, measuring the infarction size, inflammation factor, and antioxidative enzyme activities. DSY decreased the infarction size, IL-6, CRP, TNF-*α*, and MAD, as well as enhanced SOD activities and glutathione [87, 88]. These results suggested that DSY plays a remarkable role against ischemic myocardial damage in rats, probably through an anti-inflammatory reaction and antioxidative properties.

#### 2.1.3. Patented Drugs from Traditional Chinese Medicine

*Dunye Guanxinning (DG):* DG, a traditional Chinese herbal medicine formula, is extracted from the rhizomes of *Dioscorea zingiberensis* and is widely used for the treatment of angina, hyperlipidemia, and coronary heart disease [89, 90]. A study [91] explored that DG ameliorates myocardial I/R damage by suppressing caspase-1 activity and neutrophil infiltration. The result suggested that DG restrained neutrophil infiltration and decreased the interleukin-1 beta (IL-1*β*). In addition, DG suppressed caspase-1 activity and activatory AMPK phosphorylation in rat hearts. Thus, DG may be able to suppress the inflammatory response by the AMPK pathway [90, 91].

*Hongjingtian injection (HJT):* HJT is extracted from *Rhodiola rosea* and could prevent all kinds of vascular diseases like coronary heart disease and angina [92]. A study [93] assessed the cardioprotective effects of HJT. The experiments showed that HJT suppressed H/R-induced apoptosis and adjusted the expression of apoptosis-related proteins caspase 3 and Bcl-2. In addition, HJT obviously regulated the activity of the Akt, ERK/mTOR, and Akt/Beclin-1 pathways in cardiac cell autophagy. HJT prominently reduced the infarct size and ameliorated cardiac function and enhanced the light chain 3B (LC-3B) protein expression in the coronary ligation rat model. As a result, HJT reduced myocardial injury by adjusting the balance of apoptosis and autophagy and by decreasing ROS levels [94, 95].

*Guanxintai (GXT):* GXT, a Chinese compound formula, is often used in the treatment of cardiovascular diseases and is mainly composed of *Ginseng*, *Astragalus*, *Rehmannia*, *Ophiopogon root*, etc. Previous studies have verified the cardioprotective effects of GXT on the angina [96–99] and arrhythmia [100], as well as its inhibitory actions on blood lipid levels [101]. Yang et al. [102] studied the protective actions of GXT on ischemic cardiomyocytes and the related antioxidative effects. The research findings showed that GXT decreased the degree of myocardial cell injury and apoptosis and partly ameliorated cardiac function after MI. Furthermore, GXT restrained the ROS level and reduced NADPH oxidase (NOX) and mitogen-activated protein kinase (MAPK) protein expression. Therefore, the cardioprotective effects of GXT are exerted by the activity of the antioxidative NOX suppression [103, 104].

*Cardiotonic pill (CP):* CP includes *Salvia miltiorrhiza*, *Borneol*, and *Panax notoginseng* and is extensively used for the treatment of ischemic angina pectoris. A study [105] explored the underlying mechanisms of CP antioxidative activity. Male rats had left anterior descending artery ligation, and then, reperfusion was performed. The result suggested that CP decreased myocardial damage, ROS, and microcirculation disturbance. CP prominently suppressed I/R-induced NOX subunit p67phox, gp91phox, and p47phox protein expression. These data indicated that the CP alleviated I/R-induced rat myocardial damage and the disorder of microcirculation by inhibiting NOX activity [105–107].

*Shenxian-shengmai (SXSM):* SXSM oral liquid, a Chinese compound formula, has been widely used for bradyarrhythmias in clinical practice [108, 109]. MI, especially in right coronary-associated cardiac diseases, can give rise to bradyarrhythmias. A study [110] evaluated the functions of SXSM on bradyarrhythmias and cardiac insufficiency caused by myocardial I/R damage. Results showed that SXSM enhanced heart rate and protected from myocardial I/R damage. The study also discovered that SXSM ameliorated myocardial interstitial dilatation and the structural changes of myocardial cells. At the same time, SXSM protected myocardial cells against ROS induced by H_2_O_2_ and I/R damage by decreasing the intracellular levels of ROS. Furthermore, SXSM enhanced the activity of SOD and aggrandized the content of GSH by accelerating the glutamate-cysteine ligase catalytic subunit (GCLC) expression and GSH-Px activity, suggesting the antiarrhythmia and cardioprotective effects [111] (Table 1).

### 2.2. Ischemic Heart Failure

#### 2.2.1. Patented Drugs from Traditional Chinese Medicine

*Qi-shen-yi-qi (QSYQ):* QSYQ, a formula used for the routine treatment of HF in China, includes *Radix*, *Astragali mongolici*, *Salvia miltiorrhiza Bunge*, *Flos Lonicerae*, *Scrophularia*, *Radix Aconiti Lateralis preparata*, and *Radix glycyrrhizae* and has been proven to ameliorate cardiac function by downregulating the Renin-Angiotensin-Aldosterone System (RAAS) activity [112, 113]. A study [114] surveyed the treatment with QSYQ ischemic heart failure prevention by alleviating oxidative stress and suppressing inflammation. Rats were processed by coronary artery ligation, and then, the indicators of fibrosis such as Masson dyeing, matrix metalloproteinases (MMPs) and collagens, and inflammation factors were detected. The study demonstrated that QSYQ ameliorated cardiac function via reducing the degree of myocardial fibrosis, TNF-a, NF-*κ*B, and IL-6-STAT3 pathways and modulating angiotensin II-NADPH oxidase-ROS-MMP pathways [114, 115].

*Tongxinluo (TXL):* TXL is a prescription compound of Chinese medicine and has been verified as having anti-inflammatory, lipid-lowering, and antioxidant effects in ameliorating ischemic heart diseases [116]. A study [117] explored if TXL protected against the pressure overload-inducedischemic heart failure in mice. The transverse aortic constriction (TAC) operation was carried in mice to induce ischemic heart failure. TXL ameliorated cardiac function and relieved cardiac hypertrophy and myocardial fibrosis after treatment. Furthermore, TXL also enhanced myocardial capillary density and reduced oxidative stress damage by activating the vascular endothelial growth factor (VEGF)/Akt/eNOS signaling pathway [118].

*YiQiFuMai powder injection (YQFM): YQFM, a Chinese medicinal formula rediscovered on the basis of Shengmai San, is extracted from Panax ginseng, Ophiopogon japonicus, and Schisandra chinensis and is widely used to treat angina and ischemic heart failure [119, 120]. Another study [121] noticed the therapeutic effect of YQFM on coronary artery occlusion-induced ischemic heart failure. Ischemic heart failure was induced by coronary artery occlusion in mice. After treatment with YQFM, the result displayed that YQFM can reduce LDH and CK activities and levels of MDA, N-terminal pro-B-type natriuretic peptide (NT-proBNP), and hydroxyproline (HYP). Moreover, YQFM relieves coronary artery occlusion-induced ischemic heart failure by ameliorating the cardiac function and structure damage, oxidative stress, and cell apoptosis and suppressing the MAPK pathways [122–125].*

### 2.3. Angina

#### 2.3.1. Tongmai Yangxin (TMYX) pill

TMYX, a frequently used drug, is a Chinese compound formula used in the treatment of angina [126]. It mainly includes *rehmannia*, *Caulis Spatholobi*, *Ophiopogon*, *licorice*, *Polygonum multiflorum*, *donkey-hide gelatin*, *fructus schisandrae*, *Codonopsis pilosula*, *tortoise*, *dates*, and *cassia*. Metabolomics is a vital part of systems biology, which aims to monitor the changes of endogenous metabolites under physiological or pathological conditions. Cai et al. [127] analyzed the serum samples in clinical patients after oral administration of TMYX gathered from seven different clinical units in China. Using performance liquid chromatography, they tested metabolite profile changes in serum samples. Biomarkers, including metabolism, oxidative stress, and inflammation, were measured. The result indicated that after TMYX treatment, 10 biomarkers were reversed to normal conditions. These biomarkers participate mainly in energy metabolism, oxidative stress, and inflammation. As a result, TMYX has a therapeutic action via relieving myocardial energy disturbance, ROS, and inflammatory response [127–129]. The study, which is the first multicenter clinical study to reveal the basis and therapeutic mechanism of molecular biology of TMYX on the stable angina, can provide an objective index for the evaluation of the efficacy of TMYX in the stable angina pectoris, setting the stage for the clinical use of TMYX (Table 2).

### 2.4. Coronary Atherosclerotic Heart Disease

#### 2.4.1. Single Chinese Herbal Medicines

*Radix notoginseng: Radix notoginseng*, a traditional Chinese medicine extracted from the roots of *Panax notoginseng*, is widely planted and used as an herbal medicine in Southern China. It indicates multiple biological activities, and it is also used as a therapeutic agent for coronary heart disease and peroxidation [130, 131]. A study [132] explored the cardioprotection effect of *Radix notoginseng* in cardiovascular system diseases related to hyperlipidemia and excess cholesterol. The rat model was established by using a dietary supplement to keep a high fat diet. *Radix notoginseng* led to a significant reduction in cholesterol and triglycerides, with a rise in the high-density lipoprotein-cholesterol. In addition, *Radix notoginseng* ameliorated antioxidant status through the SOD and glutathione peroxidase (GPx) activity and decreased the lipid peroxidation [133, 134]. The result showed that *Radix notoginseng* could ameliorate lipid distributions, suppress peroxidation, and enhance antioxidant enzymes activity, thereby decreasing the occurrence of CHD.

*Pomegranate:* pomegranate fruit is abundant in polyphenols, has an antioxidant activity, and has been suggested to have advantageous effects in cardiovascular disease [135]. The impacts of pomegranate on ROS and inflammation in the model of coronary heart disease in mice have been studied [136, 137]. Transgenic mice were treated with pomegranate extract [138]. Pomegranate could improve cardiac enlargement and electrocardiogram (ECG) abnormalities by reducing macrophage infiltration, lipid accumulation, ROS, and monocyte chemotactic protein-1 in transgenic mice with coronary atherosclerotic plaque. These results indicated that the protective effect of pomegranate against atherosclerosis may relate to reduce inflammation and ROS.

#### 2.4.2. Patented Drugs from Traditional Chinese Medicine

*Shengmai San (SMS):* SMS includes *Panax ginseng*, *Schisandra chinensis*, and *Ophiopogon* and is a Chinese patent medicine used to treat CHD with antioxidative effects [139]. There was a study [140] which explored the influence of SMS on lipid peroxides and antioxidant reactions in the heart of cholesterol-raised rats. Antioxidant activities and ROS markers in the heart of rats were assessed. Results suggested that GSH-Px, glutathione-S-transferase (GST), and SOD activities were slightly improved after the SMS treatment [141] (Table 3).

## 3. Discussion

Awareness of the importance of ROS in CHD pathogenesis and the development of novel treatments has increased [142]. As a crucial resource of treatment, TCM has multiple bioactivities with antioxidative ability [143, 144]. As a result, we summarized the research progress of TCM on the treatment of CHD by regulating ROS.

During MI, mechanisms of pathogenesis are associated with a number of factors, like the large amount of free radical generation, enhanced inflammation, and apoptosis [145]. When MI/R injury happens, cardiac intracellular calcium overload can increase XO synthesis and NOX, resulting in a rapid increase of ROS generation [146]. OSR cardioprotection, CP, and SXSM against MI in rats were correlated with antioxidant properties, particularly regarding NOX. Meanwhile, inflammatory signaling pathways are related to the occurrence and development of CHD, and I/R injury is closely connected with increased inflammation [147]. Astragalin, Barbaloin, BXT, and DSY can act on various targets such as suppression of NOX and the rise of GSH, which efficiently decreases ROS injury after I/R damage. Moreover, the anti-inflammatory action of TCM is, at least partly, attributed to its antioxidant effects. A number of studies have also indicated that the upregulation of several antiapoptotic factors and proapoptotic genes, such as the Bcl-2 and Bax, plays a vital function in the ischemic tissue [148, 149]. Curcumin, *G. acuta*, HJT, and GXT offer cardioprotection by ameliorating heart functions, inhibiting the ROS of cardiac cells, enhancing the release of antioxidant enzymes, and restraining mitochondria disorder and cardiomyocyte apoptosis during I/R damage. There is growing evidence that the PI3K/Akt and Nrf2 pathways help in ROS resistance and play a key function in improving myocardial cell survival [150, 151]. Activating antiapoptotic signaling pathways such as Nrf2 and PI3K/Akt could adjust Bcl-2 and suppress caspase c activation. Therefore, a large number of studies have shown that TCM treatment, such as SAL, TAN, DSS, OP-D, and azafrin, could decrease cardiomyocyte ROS and apoptosis through activation of the Nrf2 and PI3K/Akt signaling pathways during I/R damage. AMPK can also upregulate the cell antioxidant enzymes such as SOD and catalase, thereby decreasing oxidative damage [152]. The activation of the AMPK signaling pathway during I/R injury has been thought to be a mechanism of treatment against ROS and myocardial damage [153]. Above all, PUN, Barbaloin, DG, and GXT have been noticed to improve mitochondrial damage and ROS by the AMPK signaling pathway.

In ischemic heart failure, the oxidative stress system is activated, thereby significantly promoting coronary arterial disease and damaging cardiac myocytes [154]. In this pathway, NOX plays an important role in the occurrence and progression of IHF [155, 156]. At the same time, increased oxidative stress combined with the activation of a variety of inflammatory and apoptosis pathways significantly influence the effect on the occurrence and development of ischemic heart failure [157]. TCM has been used to cure ischemic heart failure for thousands of years. A lot of TCMs, such as QSYQ, TXL, and YQFM, showed cardioprotection against HF by alleviation of apoptosis, inflammation, and ROS.

Increased oxidative stress, disturbed lipid metabolism, and increased inflammation are critical factors in the occurrence and development of atherosclerosis and subsequent CHD [158, 159]. Radix notoginseng, pomegranate, and SMS offer tissue damage protection, attributed to ROS, by decreasing lipid peroxidation and enhancing the activity of antioxidant enzymes. In angina, pathogenesis mainly involves energy metabolism, ROS, and inflammation [160]. TMYX may have therapeutic actions by ameliorating myocardial energy supply dysfunction and amino acid disorders and by reducing ROS and inflammation.

## 4. Conclusions

In conclusion, there is overwhelming evidence that oxidative stress is associated with the pathogenesis of CHD. TCM therapy has unique advantages in CHD. In recent years, Chinese medicine has made great progress in the treatment of CHD, which can effectively ameliorate the symptoms of patients and improve the quality of life of patients. Compared with Western medicine, it has significant therapeutic effects, few side effects, and no obvious drug dependence. The treatment of this disease by TCM has a broad prospect, and it is worthy of further promotion and development.

## Figures and Tables

**Table 1:** The role of traditional Chinese medicine (TCM) in the regulation of reactive oxygen species (ROS) in myocardial infarction (MI).

Type of TCM	TCM (molecular formula)	Type of study	Mechanism of action	References
The bioactive ingredients of TCM	SAL (C_36_H_30_O_16_; C_26_H_22_O_10_), TAN (C_18_H_12_O_3_; C_19_H_18_O_3_)	In vivo	Downregulation of factors participated in oxidative stress and apoptosis; inhibition of intracellular calcium and cell adhesion pathways	Wang et al. [129]
	Danshensu (C_9_H_10_O_5_)	In vivo	Activation of the Akt/ERK1/2/Nrf2 signaling pathway	Yu et al. [35]
	Astragalin (C_21_H_20_O_11_)	In vivo	Antiapoptotic, antioxidative, and anti-inflammatory activities	Qu et al. [41]
	OP-D (C_44_H_70_O_16_)			
	Curcumin (C_21_H_20_O_6_)	In vivo	Activating JAK2/STAT3 signal pathway, reducing oxidative damage and suppressing myocardium apoptosis	Liu et al. [111]
	Punicalagin (C_48_H_28_O_30_)	In vivo	Activation of AMPK	Ding et al. [60]
	Barbaloin (C_21_H_22_O_9_)	In vivo	Antioxidative, anti-inflammatory	Zhang et al. [93]
	OSR (C_15_H_24_N_2_O_2_)	In vivo	Antiapoptotic, anti-inflammatory, and antioxidative	Meng et al. [68]
	*G. acuta* (C_13_H_8_O_2_)	In vivo	Activities of antioxidative and antiapoptosis	Wang et al. [81]
	Azafrin (C_27_H_38_O_4_)	In vivo, in vitro	Activation of the Nrf2-ARE pathway	Yang et al. [158]

Traditional Chinese medicine decoction	Bao-Xin-Tang	In vivo	Antioxidant and anti-inflammation properties	Wang et al. [81]
	Dan-Shen-Yin	In vivo	Anti-inflammatory and antioxidant properties	Yan et al. [86]

Patented drugs from traditional Chinese medicine	Dunye Guanxinning	In vivo	Inhibits inflammasome activity through the AMPK pathway	Zhang et al. [91]
	Hongjingtian injection	In vivo, in vitro	Decreasing myocardial oxidative damage	Zhang et al. [93]
	Guanxintai	In vivo, in vitro	Reduced NOX and MAPK proteins	Yang et al. [158]
	Cardiotonic pill	In vivo	Inhibiting NOX activity	Yang et al. [105]
	Shenxian-shengmai	In vivo	Enhanced the activity of SOD and aggrandized the content of GSH	Zhao et al. [110]

TCM: traditional Chinese medicine; SAL: salvianolic acid; TAN: tanshinone; OP-D: Ophiopogonin D; OSR: oxysophoridine; *G. acuta*: *Gentianella acuta*; SOD: superoxide dismutase; GSH: glutathione; NOX: NADPH oxidase; MAPK: mitogen-activated protein kinase; AMPK: adenosine monophosphate-activated protein kinase; Nrf2: nuclear factor erythroid-2-related factor 2; Akt: serine/threonine kinase.

**Table 2:** The role of traditional Chinese medicine (TCM) in the regulation of reactive oxygen species (ROS) in ischemic heart failure and angina.

Type of disease	Type of TCM	TCM (molecular formula)	Type of study	Mechanism of action	References
Ischemic heart failure	Patented drugs from traditional Chinese medicine	Qi-shen-yi-qi	In vivo	Recovering angiotensin II-NADPH oxidase-ROS-MMP pathways	Li et al. [114]
		Tongxinluo	In vivo	Activation of the VEGF/Akt/eNOS signaling pathway	Wang et al. [117]
		YiQiFuMai powder injection	In vivo	Ameliorating cardiac function and structure damage, oxidative stress, and cell apoptosis and inhibiting the MAPK signaling pathways	Pang et al. [121]
Stable angina		Tongmai Yangxin pill	In clinic	Attenuating oxidative stress and inflammation	Cai et al. [127]

ROS: reactive oxygen species; MMPs: matrix metalloproteinases; VEGF: vascular endothelial growth factor; Akt: serine/threonine kinase; eNOS: endothelial nitric oxide synthase; MAPK: mitogen-activated protein kinase.

**Table 3:** The role of traditional Chinese medicine (TCM) in the regulation of reactive oxygen species (ROS) in coronary atherosclerotic heart disease.

Type of TCM	TCM (molecular formula)	Type of study	Mechanism of action	References
Single Chinese herbal medicines	Radix notoginseng	In vivo	Inhibit peroxidation and increase the activity of antioxidant enzymes	Xia et al. [132]
	Pomegranate	In vivo	Reduced oxidative stress and inflammation	Al-Jarallah et al. [138]
Patented drugs from traditional Chinese medicine	Shengmai San	In vivo	Inhibit peroxidation and increase the activity of antioxidant enzymes	Yao et al. [140]
